# BDNF Val66met Gene Polymorphism in Primary Acute and Subacute Stroke Functional Recovery: A Systematic Review

**DOI:** 10.3390/biomedicines14071637

**Published:** 2026-07-20

**Authors:** Juliana Moura Alves Seixas, Cristina Lemos Barbosa Furia, Matheus Gomes de Castro, Larissa Sousa Silva Bonasser, Ligia Canongia de Abreu Cardoso Duarte, Calliandra Maria de Souza Silva, Izabel Cristina Rodrigues da Silva

**Affiliations:** 1 Postgraduate Program in Health Sciences and Technologies, Faculty of Health Sciences and Technologies, University of Brasília (UnB), Brasília 72220-900, Brazil; juliana.seixas@aluno.unb.br (J.M.A.S.); matheuscastrodf@gmail.com (M.G.d.C.); ligia.canongia@gmail.com (L.C.d.A.C.D.); cdssilva@gmail.com (C.M.d.S.S.); 2 Clinical Analysis Laboratory, Molecular Pathology Sector, Pharmacy Department, Faculty of Health Sciences and Technologies, University of Brasília (UnB), Brasília 72220-900, Brazil; 3 Faculty of Health Sciences and Technologies, University of Brasília (UnB), Brasília 72220-900, Brazil; furiacristina@gmail.com; 4 Postgraduate Program in Health Sciences, University Campus Darcy Ribeiro, University of Brasília (UnB), Brasília 72220-900, Brazil; 5 Academic Unit of Biotechnology Engineering (UAEB), Center for Sustainable Development of the Semi-Arid Region (CDSA), Sumé Campus, Federal University of Campina Grande (UFCG), Sumé-Paraiba 58540-000, Brazil

**Keywords:** stroke, brain-derived neurotrophic factor, BDNF Val66Met, rs6265, polymorphism genetic, recovery of function, single-nucleotide variant

## Abstract

**Background/Objectives**: Stroke remains a leading global cause of death and disability, resulting from acute focal injury to the central nervous system. The brain-derived neurotrophic factor (BDNF) gene has been extensively investigated for its involvement in post-stroke neuroplasticity and recovery. This systematic review examines the influence of the BDNF Val66Met (rs6265) variant on functional recovery during the acute and early subacute phases of primary stroke. **Methods**: The review followed PRISMA guidelines and was registered in PROSPERO (CRD42024549967). Comprehensive literature searches were performed in PubMed, Web of Science, and the Virtual Health Library in December 2024 to identify observational and interventional studies involving adults with acute or subacute primary stroke that evaluated this polymorphism. **Results**: Of 475 records identified, 11 studies met the inclusion criteria. Sample sizes ranged from 14 to 829 participants, with follow-up intervals ranging from 2 days to 3 months post-stroke. All studies included ischemic stroke, and seven also included hemorrhagic stroke cases. Outcomes assessed were motor recovery, cortical excitability, swallowing, and language function. Overall, most studies reported poorer recovery among Met allele carriers (Val/Met or Met/Met), particularly in motor outcomes. Evidence for cortical excitability and swallowing also suggested unfavorable effects, while language outcomes were inconclusive. Methodological heterogeneity contributed to inconsistent findings. **Conclusions**: The Met carriers were the most prevalent and frequently associated with poorer post-stroke outcomes, though results varied. These findings underscore the complex interplay between genetics and post-stroke recovery and indicate the need for more robust, standardized research across diverse populations.

## 1. Introduction

Stroke is defined as a neurological deficit resulting from an acute focal lesion of the central nervous system. The majority of cases are ischemic, caused by reduced cerebral blood flow, whereas hemorrhagic strokes, resulting from arterial rupture, occur less frequently [1,2]. Clinically, the course of stroke can be categorized into distinct temporal phases: hyperacute (0–24 h), acute (1–7 days), early subacute (7 days to 3 months), late subacute (3–6 months), and chronic (>6 months) [3].

Stroke is the second leading cause of death and the third leading cause of combined death and disability worldwide [4]. Most survivors experience impairments in motor, sensory, swallowing, cognitive, emotional, and speech functions [5,6]. Functional recovery occurs heterogeneously, with different deficits showing distinct magnitudes and trajectories over time, reflecting variations in neural plasticity across brain systems after stroke [7]. Evidence indicates that the greatest degree of recovery occurs between 1 and 6 months following the event [8].

Multiple genes contribute to brain plasticity and adaptive responses. Brain-derived neurotrophic factor (BDNF), among the most extensively studied neurotrophins in the central nervous system, plays a crucial role in regulating cellular processes necessary for brain development and maintenance [9]. BDNF is therefore a key mediator of post-stroke recovery, influencing neuronal growth, survival, and synaptic differentiation and plasticity [7,10,11]. The effect of BDNF on recovery outcomes is modulated by factors such as time since stroke, lesion characteristics, and patient age, indicating its potential as a genetic factor underlying the heterogeneity observed in stroke rehabilitation [7,10].

The BDNF gene, located on chromosome 11p14.1, contains the functional single-nucleotide variant (SNV) rs6265, which results in a valine (G) to methionine (A) substitution at codon 66 (Val66Met) of the BDNF precursor (pro-BDNF) [12,13,14]. This variant is associated with reduced BDNF protein secretion and may lead to lower activity-dependent BDNF release [13], potentially impacting post-stroke recovery.

A comprehensive understanding of BDNF genetics and its regulatory mechanisms may facilitate improved post-stroke patient management by supporting personalized rehabilitation strategies, potentially enhancing recovery times and reducing healthcare costs [15]. This systematic review evaluated the impact of the Val66Met (rs6265) variant on functional recovery after stroke by comparing the frequency of A (Met) carriers with GG (Val/Val) carriers in patients during the acute and early subacute phases, defined as up to 3 months post-stroke.

## 2. Materials and Methods

### 2.1. Search Strategy and Selection Criteria

This systematic review followed the Preferred Reporting Items for Systematic Reviews and Meta-Analyses (PRISMA) guidelines (PROSPERO CRD42024549967). Inclusion criteria were defined using the Population, Exposure, Comparison, Outcome, and Study Type (PECOS) framework: (1) Population: human participants with primary stroke in the acute and subacute phase; (2) Exposure: BDNF Val66Met genetic variant; (3) Comparison: GG (Val/Val) genotype versus Met carriers (Val/Met + Met/Met); (4) Outcome: functional recovery outcomes according to BDNF Val66Met genotype; (5) Study Type: observational and interventional studies reporting genotypic frequencies of the BDNF Val66Met variant in acute stroke and describing laboratory methods according to eligibility criteria.

The inclusion criteria were as follows: patients with primary stroke (ischemic or hemorrhagic). Primary stroke was defined as a first-ever cerebrovascular event, excluding cases of recurrent stroke; acute and/or early subacute phase (up to 3 months after the stroke); participants aged 18 years or older, male or female; open-access observational or interventional studies that described the Val66Met (rs6265) genetic variant genotype frequencies and presented laboratory methods. However, animal studies, studies with incomplete data (including statistical data), reviews, meta-analyses, and abstracts were excluded from the analysis.

In December 2024, a comprehensive search was conducted using the Web of Science, PubMed (MEDLINE), and Virtual Health Library (VHL) databases. Initially, no filters were applied. Due to the large volume of publications, the search was subsequently limited to articles involving acute and subacute primary stroke populations, with no restriction on publication year. Embase/Scopus were not included; however, the selected databases are considered sufficiently comprehensive to capture the relevant literature.

The search terms included “BDNF,” “polymorphism OR variant,” and “stroke,” as defined by the Medical Subject Headings (MeSH) vocabulary. These terms were combined using the Boolean operator “AND.” In addition, a comprehensive search strategy was applied using synonymous terms and specific genetic variants, structured as follows: (“BDNF” OR “brain-derived neurotrophic factor”) AND (“Val66Met” OR “rs6265”) AND (“stroke” OR “ischemic stroke”) AND (“recovery” OR “rehabilitation”).

### 2.2. Study Selection and Data Extraction

Two reviewers (JS and CS) collaborated on the article selection in two phases. In the first phase, each reviewer independently reviewed the title and abstract of each article, verifying its eligibility according to the PECOS strategy. The Rayyan tool, developed by the Qatar Computing Research Institute (QCRI), assisted in this initial analysis and removed duplicates. In the second phase, the same two reviewers (JS and CS) independently reviewed the full texts of the preselected articles, consistently applying the established eligibility criteria.

In both phases, disagreements or questions were discussed between the two reviewers. If an agreement could not be reached, a third reviewer (IS) was consulted. The two reviewers (JS and CS) independently extracted predefined data into an electronic spreadsheet using Microsoft Office Excel. The extracted data included: author, study title, objective, year of publication, country of study, participants, sample size, stroke pathophysiology (ischemic and/or hemorrhagic), scales used, stroke onset/follow-up time, genotypic frequencies of the Val66Met (rs6265) genetic variant, laboratory methodology, primary outcome, functional outcomes, and *p*-value.

### 2.3. Bias Risk in Each Study

The risk of bias of each study was assessed using the Genetic Risk Prediction Studies (GRIPS) guideline, which comprises 25 items designed to promote the selection of high-quality studies and facilitate the comparison and application of data from studies with different designs, methods, or analyses [16]. Two reviewers (JS and CS) independently examined the presence or absence of these items to assess the methodological quality, results, and discussion of the included articles. In cases of disagreement, a third reviewer (IS) was contacted. Studies that presented 75% or more of the items were considered to be of good quality.

Due to substantial heterogeneity in outcome measures, study designs, and assessment timepoints, a formal quantitative meta-analysis was not performed. Instead, a structured qualitative synthesis was conducted.

## 3. Results

### 3.1. Search, Selection, and Quality Assessment of Articles

The systematic search strategy across PubMed (MEDLINE), Web of Science, and the Virtual Health Library yielded 475 records. After removing duplicate entries, 301 unique articles remained for title and abstract screening. These records were independently evaluated by two reviewers according to the predefined PECOS eligibility criteria. Following the initial screening phase, 69 studies were considered potentially eligible and underwent full-text assessment. During this stage, articles were excluded for reasons including: evaluation of chronic stroke populations beyond the predefined temporal window; focus on post-stroke depression or cognitive impairment rather than functional recovery; lack of reporting of genotype frequencies for the BDNF Val66Met polymorphism; or incomplete methodological data.

**Figure 1:**
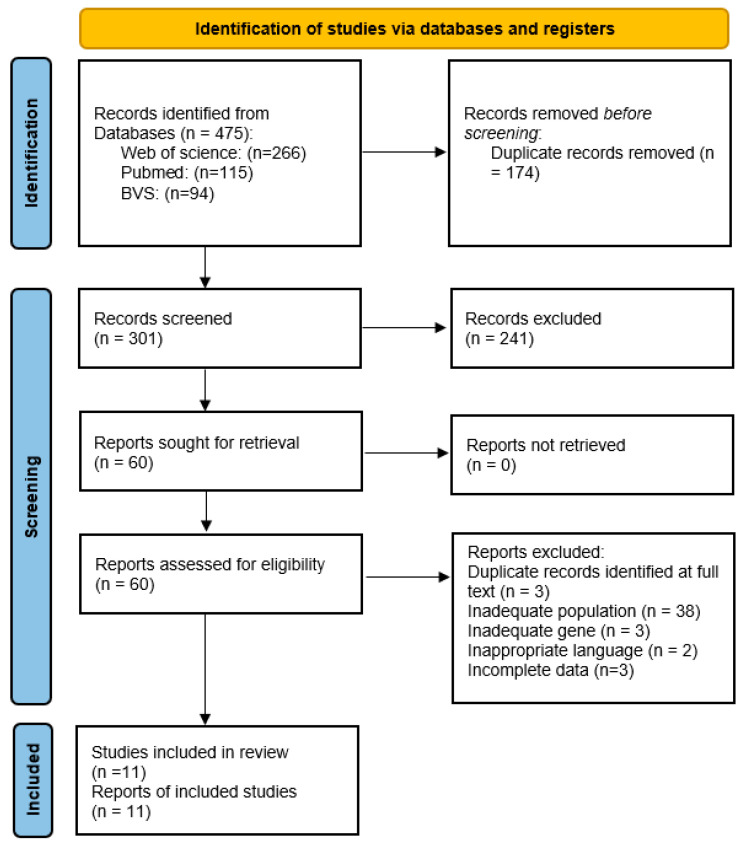
Flowchart outlining the steps adopted in the selection of articles.

After rigorous application of all inclusion and exclusion criteria, 11 studies met eligibility requirements and were included in the qualitative synthesis. The complete selection process, including the number of records identified, screened, excluded, and finally included, is detailed in Figure 1, which presents the PRISMA flow diagram summarizing the study selection pathway. Excluded studies and the specific reasons for exclusion at the full-text assessment stage are comprehensively presented in Supplementary Table S2.

### 3.2. General Characteristics of the Selected Studies

The principal characteristics of the included studies are summarized in Table 1. The eleven eligible studies were published between 2012 and 2021. Most investigations were conducted in South Korea, while the remaining studies were carried out in Poland, the United States, and Italy. This geographic distribution reflects a predominance of East Asian cohorts, with limited representation from Western populations.

Sample sizes varied substantially, ranging from 14 to 829 participants. The interval between stroke onset and functional assessment spanned from 2 days to 3 months, consistent with the predefined acute and early subacute phases adopted in this review.

All included studies evaluated individuals with ischemic stroke. Seven investigations also included patients with hemorrhagic stroke [17,18,19,20,21,22]. Participant age distributions varied across cohorts; while most studies included middle-aged and older adults, one large cohort specifically examined younger stroke patients.

The functional domains assessed encompassed motor recovery, cortical excitability, swallowing function (dysphagia), and language performance (aphasia). A variety of validated scales and instruments were employed, including the Fugl–Meyer Assessment (FMA), modified Rankin Scale (mRS), National Institutes of Health Stroke Scale (NIHSS), Barthel Index, and Functional Oral Intake Scale (FOIS). Several studies incorporated complementary neurophysiological and neuroimaging measures, such as transcranial magnetic stimulation (TMS) and diffusion tensor imaging (DTI), to evaluate cortical plasticity and white matter integrity.

The diversity of outcome measures, assessment timepoints, and methodological approaches underscores the heterogeneity present across studies, as detailed in Table 1.

Table 2 presents study-level comparisons, including objectives, sample sizes, genotype distributions, statistical significance, *p*-values, and effect directions. The table shows variability in methodology and outcomes, with several studies reporting unfavorable results for Met allele carriers. Heterogeneity in outcome measures and study designs prevented quantitative pooling of effect sizes.

A structured synthesis of directional findings across domains is presented in Table 3, which summarizes the number of studies investigating each recovery domain, the cumulative number of participants represented, and the proportion of investigations reporting statistically significant associations between the Met allele and poorer functional outcomes.

In the motor recovery domain, the majority of studies reported significantly poorer recovery trajectories among Met allele carriers, including assessments of upper-limb motor function, global disability indices, and corticospinal tract integrity. These findings were observed across both observational and interventional designs, including studies using repetitive transcranial magnetic stimulation.

Evidence on cortical excitability and swallowing function was limited to single-cohort studies, both of which reported statistically significant genotype-associated differences. Language recovery was examined in one study, which did not identify a statistically significant association between genotype and early aphasia outcomes.

**Table 1:** Characteristics of studies included in the systematic review.

Author	Title	Year	Country	Population Sample (*n*)	Stroke Type	Admission Time and Intervention Time	What Was Evaluated?	Scales Used
Mirowska-Guzel et al. [23]	Association between BDNF-196 G > A and BDNF-270 C > T polymorphisms, BDNF concentration, and rTMS-supported long-term rehabilitation outcome after ischemic stroke.	2012	Poland	*n* = 46 (M: 29; F: 17) Age: 62.09 ± 10 Aphasia: 20 Hemiparesis: 26	Ischemic: 46	3 months (T0) 3 weeks of intervention	rTMS effect on post-stroke hemiparesis or hand aphasia	FMA; ASRS
Kim et al. [17] *	Effect of the presence of brain-derived neurotrophic factor val66met polymorphism on the recovery in patients with acute subcortical stroke.	2013	Republic of Korea	*n* = 14 (M: 7; F: 7) Groups: Met carrier: 7 Age: 69.0 ± 13.8 Val/Val: 7 Age: 65.0 ± 7.8	Ischemic: 12 Hemorrhagic: 2	>10 days (T0); 1 month (T1); 3 months (T2)	BDNF Val66Met effect on functional recovery after subcortical stroke	mRS; FMA; NIHSS; K-MBI; K-MMSE; MEP
Chang et al. [18] **	BDNF polymorphism and differential rTMS effects on motor recovery of stroke patients.	2014	Republic of Korea	*n* = 44 (M: 25; F: 19) Groups: Met carrier: 35 Age: 53.4 ± 13.7 Val/Val: 9 Age: 58.4 ± 9.6	Ischemic: 32 Hemorrhagic: 12	<2 weeks (T0) Evaluated immediately after (Post-rTMS) and 2 months after (Follow-up) the intervention	rTMR effect on motor recovery	FMA; BBT
Di Lazzaro et al. [24]	Val66Met BDNF gene polymorphism influences human motor cortex plasticity in acute stroke.	2015	Italy	*n* = 20 (M: 11; F: 9) Age: 64.15 ± 2.4	Ischemic: 20	<10 days (T0)	BDNF Val66Met effects on changes in human brain excitability	NIHSS; TMS; MEP; TMR; AMT
Kim et al. [19]	The brain-derived neurotrophic factor Val66Met polymorphism and degeneration of the corticospinal tract after stroke: a diffusion tensor imaging study	2016	Republic of Korea	*n* = 35 (M: 18; F: 17) Age: 53.52 ± 4.83 Control group: 23 (M: 10; F: 13) Age: 53.52 ± 4.83	Ischemic: 24 Hemorrhagic: 11	2 weeks (T0); 1 month (T1); 3 months (T2)	BDNF Val66Met effect on corticospinal tract degeneration and motor recovery	FMA; DTI; CST
Chang et al. [25] ***	Factors influencing the response to high-frequency repetitive transcranial magnetic stimulation in patients with subacute stroke	2016	Republic of Korea	*n* = 62 (M: 32; F: 30) Age: 56.7 ± 13.0 Good responders: 20 Poor responders: 42	Ischemic: 42 Hemorrhagic: 20	<4 weeks (T0) 10 rTMS sessions of the primary motor cortex of the affected hemisphere over a period of 2 weeks	Factors influencing rTMR effect on motor recovery	FMA; CST; DTT; MEP
Chang et al. [20] ****	Association Between Brain-Derived Neurotrophic Factor Genotype and Upper Extremity Motor Outcome After Stroke	2017	Republic of Korea	*n* = 97 (M: 53; F: 44) Age: 57.9 (29–80) Without severe motor impairment: 31 With severe motor impairment: 66	Ischemic: 70 Hemorrhagic: 27	<4 weeks (T0); 3 months (T1) 2-week rehab program	Potential prognostic role of BDNF genotype in upper limb motor outcome	FMA; CST; MEP; DTT
Park et al. [26]	Differential Relationship between Microstructural Integrity in White Matter Tracts and Motor Recovery following Stroke Based on Brain-Derived Neurotrophic Factor Genotype	2020	Republic of Korea	*n* = 58 (M: 48; F: 10) Groups: Met carrier: 41 Age: 62.1 ± 11.7 Val/Val: 17 Age: 64.7 ± 12.4	Ischemic: 58	2 weeks (T0); 3 months (T1)	Relationship between white matter integrity and motor recovery by genotype	FMA; DTI; CST; MEP
Bembenek et al. [21]	Prediction of Recovery and Outcome Using Motor Evoked Potentials and Brain-Derived Neurotrophic Factor in Subacute Stroke	2020	Poland	*n* = 71 (M: 45; F: 26) Age: 65.32 ± 11.8	Ischemic: 68 Hemorrhagic: 3	2–14 days (T0); 30 days (T1); 90 days (T2)	Prognostic value of MEP elicited by TMS, serum BDNF and genotype in the hand paresis recovery	NIHSS; MRC; MBS; mRS; BI; MEP; CMCT; RMT
Braun et al. [27]	Effects of the BDNF Val66Met polymorphism on functional status and disability in young stroke patients	2020	United States of America	*n* = 829 (M: 482; F: 347) Age: 41.5 ± 6.9	Ischemic: 829	Hospital discharge (T0)	Effects of the Val66Met polymorphism on post-stroke functional outcomes.	mRS
Oh et al. [22] *****	Role of rs6265 BDNF polymorphisms and post-stroke dysphagia recovery-A prospective cohort study.	2021	Republic of Korea	*n* = 206 (M: 136; F: 66) Age: 63.8 ± 11.6 Good outcome: 126 Poor outcome: 86	Ischemic: 119 Hemorrhagic: 81 Mixed: 6	2 weeks (T0); 3 months (T2)	Dysphagia recovery according to BDNF Val66Met	FOIS; MASA; MBSimP; PAS; EAT-10; NIHSS; mRS; mBI

Abbreviations: n—number; F—female; M—male; BDNF—Brain-Derived Neurotrophic Factor; Val/Val—Homozygous for Valine allele; Met carrier—Heterozygous or homozygous for Methionine allele; AMT—Active Motor Threshold; ASRS—Aphasia Severity Rating Scale; BI—Barthel Index; BBT—Box and Block Test; CST—Corticospinal Tract Integrity (ipsilesional); DTI—Diffusion Tensor Imaging Data Acquisition; DTT—Diffusion Tensor Tractography; FMA—Fugl–Meyer Assessment; FMA-UL—Fugl–Meyer Assessment—Upper Limb subscale; K-MBI—Brunnstrom Stage, Korean version Modified Barthel Index; K-MMSE—Korean version of the Mini-Mental State Examination; MBSimP—Modified Barium Swallow Impairment Profile; mBI—modified Barthel Index; MEP—Motor Evoked Potential (threshold and amplitude); rTMS—Repetitive Transcranial Magnetic Stimulation; NIHSS—National Institutes of Health Stroke Scale; mRS—Modified Rankin Scale; MRC—Medical Research Council Scale; MBS—Modified Brunnstrom Scale; CMCT—Central Motor Conduction Time; FOIS—Functional Oral Intake Scale; MASA—Mann Assessment of Swallowing Ability; PAS—Penetration–Aspiration Score; EAT-10—Eating Assessment Tool; RMT—Resting Motor Threshold; TMS—Transcranial magnetic stimulation. * Kim et al., 2013 [17]: 36 patients were recruited and completed the 3-month assessment. Patients were matched 1:1 by stroke type. Matching was performed by a blinded researcher, with one group being the MET+ allele and the other the MET− allele. As a result of the matching, seven patients in each group were selected for analysis. ** Chang et al., 2014 [18]: 47 stroke patients with hemiparesis were recruited according to the inclusion criteria. Three patients withdrew during the experimental procedure for various personal reasons, leaving 44 patients who completed the study. *** Chang et al., 2016 [25]: 64 patients were recruited; one patient withdrew from the study due to headache and another due to discomfort, although in both cases, the adverse effects disappeared soon after stopping a given set of rTMS. **** Chang et al., 2017 [20]: Of the first 103 patients, three were unable to complete the study follow-up for personal reasons, and another three were excluded from the analysis due to motion artifacts in the diffusion tensor imaging. Therefore, 97 patients with a complete data set at the 3-month follow-up (T1) were analyzed. ***** Oh et al.; 2021 [22]: Of the 218 patients initially included in this study, four were lost to follow-up, one died, and 7 refused blood collection. A total of 206 individuals were included and had complete medical records up to 3 months after stroke onset. Note: T = assessment time after stroke, as described in each article. T0: admission after stroke.

**Table 2:** Comparative results of studies evaluating the BDNF Val66Met (rs6265) polymorphism in acute and subacute primary stroke.

Author	Title	Objective	Results	Genotypic Frequency	Statistical Significance
Mirowska-Guzel et al. [23]	Association between BDNF-196 G > A and BDNF-270 C > T polymorphisms, BDNF concentration, and rTMS-supported long-term rehabilitation outcome after ischemic stroke.	Determine the allelic and genotypic distribution of BDNF-196 G > A and -270 C > T polymorphisms and evaluate the impact of repetitive transcranial magnetic stimulation (rTMS) on serum BDNF concentrations measured before rehabilitation, after the first 6 h of rehabilitation and after 3 weeks of rehabilitation.	Genotype distribution did not differ significantly between patients with hemiparesis and aphasia or between those who improved and those who did not. Furthermore, none of the investigated BDNF polymorphisms influenced serum BDNF levels. Additionally, no association was found between the BDNF-196 G > A variants and post-stroke rehabilitation effectiveness, defined as improvement on any assessed functional scale.	All: Val/Val: 35 (76%) Met carrier: 11 (24%) Aphasia: Val/Val: 17 (85%) Met carrier: 3 (15%) Hemiparesis: Val/Val: 18 (69%) Met carrier: 8 (31%).	*p* > 0.05
Kim et al. [17]	Effect of the presence of brain-derived neurotrophic factor val66met polymorphism on the recovery in patients with acute subcortical stroke.	Investigate the effect of the BDNF Val66Met polymorphism on recovery after subcortical stroke using the modified Rankin Scale (mRS).	Patients with at least one BDNF Val66Met polymorphism were compared to controls without the variant, matched for initial stroke severity, location, and type. The Val/Val group showed significant improvement in mRS at 1 and 3 months post-discharge, suggesting the Val66Met polymorphism may be associated with poorer recovery after subcortical stroke.	Val/Val: 7 (50%) Met carrier: 7 (50%)	*p* < 0.05
Chang et al. [18]	BDNF polymorphism and differential rTMS effects on motor recovery of stroke patients.	Investigate whether BDNF polymorphism influences the rTMS effect on motor recovery in patients with stroke.	No significant differences were observed in the basal motor functions of the affected upper limb and hand between the Val/Val groups and Met allele carriers.	Val/Val: 9 (20.5%) Met carrier: 35 (79.5%)	baseline *p* > 0.05
			Both groups improved immediately and at 2 months after rTMS. However, the Val/Val group had greater improvements in FMA-UL (*p* < 0.05) and BBT at follow-up, indicating a more sustained benefit than the Met allele carrier group.		*p* < 0.05 (follow-up)
Di Lazzaro et al. [24]	Val66Met BDNF gene polymorphism influences human motor cortex plasticity in acute stroke.	Evaluate motor cortex excitability to single-pulse TMS and LTP-like activity induced by a repetitive TMS (rTMS) paradigm, known as intermittent theta burst stimulation (iTBS), in acute stroke patients and correlate the electrophysiological findings with BDNF genotype.	The BDNF Val66Met polymorphism was associated with a ninefold reduction in interhemispheric imbalance in cortical excitability, as measured by RMT between HA and HU. Patients without the polymorphism showed greater imbalance than those with it.	Val/Val: 12 (60%) Met carrier: 8 (40%)	*p* = 0.036
Kim et al. [19]	The brain-derived neurotrophic factor Val66Met polymorphism and degeneration of the corticospinal tract after stroke: a diffusion tensor imaging study	Evaluate BDNF Val66Met potential role in the pathogenesis and treatment of stroke, and examine the relevance of the variant to long-term stroke outcomes, specifically regarding alterations in corticospinal tract integrity.	Met allele carriers had poorer motor outcomes than Val allele carriers at 1 month (T2) and 3 months (T3) after onset.	Val/Val: 10 (28.6%) Met carrier: 25 (71.4%)	*p* < 0.05
Chang et al. [25]	Factors influencing the response to high-frequency repetitive transcranial magnetic stimulation in patients with subacute stroke	Identify the main factors influencing the effectiveness of high-frequency rTMS to improve motor function in patients with subacute stroke and moderate to severe upper limb motor impairment.	Good responders had a significantly higher proportion of BDNF Val/Val genotypes than poor responders. Post-rTMS FMA-UL and FMA-T scores were also significantly higher in good responders.	Val/Val: 12 (19.4%) Met carrier: 50 (80.6%) Good responders (20): Val/Val: 7 (35%) Met carrier: 13 (65%) Bad responders (42): Val/Val: 5 (11.9%) Met carrier: 37 (88.1%)	*p* = 0.043
Chang et al. [20]	Association Between Brain-Derived Neurotrophic Factor Genotype and Upper Extremity Motor Outcome After Stroke	Identify prognostic factors, including intrinsic genetic factors, for upper limb motor outcome in patients with subacute stroke.	Among patients with severe motor impairment, a higher number of Met alleles independently predicted worse motor recovery at 3 months.	Val/Val: 21 (21.6%) Met carrier: 76 (78.4%) No severe motor impairment (31): Val/Val: 7 (22.6%) Met carrier: 24 (77.4%) With severe motor impairment (66): Val/Val: 14 (21.2%) Met carrier: 52 (78.8%)	*p* = 0.01
Park et al. [26]	Differential Relationship between Microstructural Integrity in White Matter Tracts and Motor Recovery following Stroke Based on Brain-Derived Neurotrophic Factor Genotype	Use FA to assess the functional role of three motor-related white matter tracts, according to BDNF genotype, in motor recovery in subacute stroke: the first consists of the majority of fibers from M1 to the medulla oblongata (M1), the second is the intrahemispheric connection from M1 to the ventral premotor cortex (M1PMv), and the third is the interhemispheric connection between bilateral M1s (CC). For each BDNF genotype, investigate the relationship between motor impairment and tract-related FA of these white matter tracts.	Val/Val genotype carriers experienced a greater reduction in FA in the ipsilesional M1–PMv tract between 2 weeks and 3 months post-stroke compared to Met allele carriers. In the Val group, FA of the contralesional M1–PMv was negatively correlated with FMA-EU at T2, while in the Met group, FA of the ipsilesional CST and corpus callosum was positively correlated with FMA-EU.	Val/Val: 17 (29.3%) Met carrier: 41 (70.7%)	*p* < 0.05
Bembenek et al. [21] †	Prediction of Recovery and Outcome Using Motor Evoked Potentials and Brain-Derived Neurotrophic Factor in Subacute Stroke	Investigate whether MEPs elicited by transcranial magnetic stimulation (TMS), serum BDNF levels, and their genotype have prognostic value for stroke recovery in patients with hand paresis.	No correlation was found between BDNF levels or their polymorphisms and motor impairment or stroke outcome. Additionally, no correlation was observed between any BDNF gene polymorphism and the severity of upper limb paresis or patient dependence after 3 months.	Met/Met: 50 (72.5%)	*p* > 0.05
Braun et al. [27]	Effects of the BDNF Val66Met polymorphism on functional status and disability in young stroke patients	Examine the effects of the Val66Met polymorphism on post-stroke functional outcomes in early-onset ischemic stroke patients aged 15–49 years.	The Met allele was significantly associated with worse outcomes on the mRS scale, conferring a higher risk of unfavorable results.	Val/Val: 664 (80.1%) Met carrier: 165 (19.9%)	*p* = 0.05
Oh et al. [22]	Role of rs6265 BDNF polymorphisms and post-stroke dysphagia recovery-A prospective cohort study.	Investigate whether genetic polymorphisms could influence the outcome of post-stroke dysphagia.	Recovery of NPM status within the first month was significantly greater in the Val/Val group than in Met allele carriers. The Val/Val group also showed greater improvement in FOIS scores, and at 3 months, demonstrated significantly greater improvement in MBSImP scores compared to the Met group. Patients with the Val/Val BDNF genotype showed faster and more significant improvement than those with the Met allele.	Val/Val: 67 (32.5%) Met carrier: 139 (67.5%)	*p* < 0.05

Note: n: number; F: female; M: male; Val (valine): G allele; Met (methionine): A allele; AH: affected hemisphere; UH: unaffected hemisphere; BDNF: brain-derived neurotrophic factor; ASRS: Aphasia Severity Rating Scale; rTMS: repeated transcranial magnetic stimulation; TMS: transcranial magnetic stimulation; FMA-UL: Fugl–Meyer assessment; BBT: box and block test; NPM: nothing orally; CST: corticospinal tract; DTI: diffusion tensor imaging; FA: fractional anisotropy; AD: axial diffusivity; RD: radial diffusivity; ADM: abductor digiti minimi muscle; mRS: modified Rankin scale; TMS: transcranial magnetic stimulation; NIHSS, National Institutes of Health Stroke Scale; MRC: Medical Research Council; MBS: modified Brunnstrom scale; BI: Barthel index; MEPs: Motor evoked potentials; † One study reported BDNF Val66Met genotype data stratified as Met/Met and Val carriers. In addition, the genotype distribution was incompletely reported, as the numbers of Val/Met and Val/Val genotypes were not explicitly provided and did not sum to the total sample size. Therefore, reclassification into Met carrier, as adopted by the other studies, was not possible, and the original categorization was maintained.

**Table 3:** Summary of directional associations between BDNF Val66Met (rs6265) polymorphism and early functional recovery after primary stroke.

Functional Domain	Number of Studies	Studies Reporting Worse Outcomes in Met Carriers	Studies Reporting No Significant Association	Overall Interpretation * (Directional Consistency)
Motor Recovery	8	6	2	Predominantly unfavorable for Met *
Cortical Excitability	1	1	0	Unfavorable for Met
Swallowing Function (Dysphagia)	1	1	0	Unfavorable for Met
Language Function (Aphasia)	1	0	1	Inconclusive
Overall	11	8	2	Predominantly unfavorable for Met

Note: * “Unfavorable for Met” indicates poorer recovery outcomes among Met allele carriers compared to Val/Val individuals. One study demonstrated genotype-associated differences in white matter integrity without direct inferiority on clinical scales.

## 4. Discussion

Brain-derived neurotrophic factor (BDNF) and its single-nucleotide variant (SNV) rs6265 are recognized as significant contributors to post-stroke recovery [15]. This review examined multiple aspects of post-stroke recovery, including motor recovery, improvements in dysphagia and aphasia, brain excitability, and white matter integrity. The findings indicate that the MET allele is frequently associated with worse outcomes [17,18,19,20,22,24,25,26,27]. However, some studies, such as Bembenek et al. [21] and Mirowska-Guzel et al. [23], reported no significant association.

Across the 11 included investigations, a directional pattern emerged regarding the association between the BDNF Val66Met polymorphism and early post-stroke recovery. Eight studies (72.7%) reported statistically significant associations between the Met allele and poorer outcomes [17,18,19,20,22,24,25,26,27]. These associations included reduced functional improvement, slower recovery trajectories, diminished responsiveness to neuromodulatory interventions, and unfavorable neuroplastic changes.

Taken together, these findings suggest that the Met allele is predominantly associated with diminished recovery efficiency and altered neuroplastic mechanisms. Although methodological heterogeneity precluded quantitative meta-analysis, the cumulative evidence indicates a deleterious directional trend during the acute and early subacute phases of stroke recovery.

### 4.1. Domain-Specific Framing

To provide a structured interpretation, findings were analyzed across functional domains. Post-stroke rehabilitation is inherently multidimensional, involving motor, bulbar, cortical, and language networks. Motor networks (primary motor cortex, premotor and supplementary motor areas, basal ganglia, cerebellum) support voluntary movement; somatosensory networks enable sensorimotor integration; language networks (Broca’s, Wernicke’s, arcuate fasciculus) underpin communication; swallowing networks integrate cortical and brainstem structures; and frontoparietal, limbic, and default mode networks contribute to attention, compensatory learning, and emotional regulation. Recovery outcomes depend on distributed plasticity across these interconnected systems.

Genetic factors, including the Val66Met polymorphism, appear to modulate variability in recovery trajectories and responsiveness to rehabilitation [17]. This variant has been linked to reduced activity-dependent BDNF secretion, which may impair synaptic potentiation and limit adaptive neuroplasticity [24]. Table 2 provides study-level comparisons, highlighting methodological variability and the predominance of unfavorable outcomes among Met carriers. Table 3 consolidates domain-level findings.

#### 4.1.1. Motor Recovery

Stroke remains a global public health concern, ranking as the third leading cause of combined disability worldwide [4]. Motor function, defined as the voluntary control of movement and muscle coordination, is frequently compromised after a cerebrovascular event [28]. Hemiplegia, one of the most prevalent sequelae, causes significant upper-limb dysfunction [19]. Most individuals with stroke experience motor deficits in the upper limbs. Repetitive transcranial magnetic stimulation (rTMS) has emerged as an intervention capable of modulating cortical excitability, promoting neuroplasticity, and contributing to functional recovery in the post-stroke period [6].

Motor recovery was the most extensively investigated domain, with eight of the eleven eligible studies assessing motor-related outcomes in a cumulative sample of 1210 participants during the acute and early subacute phases of stroke. These investigations encompassed both observational and interventional designs, employing standardized instruments such as the Fugl–Meyer Assessment (FMA), modified Rankin Scale (mRS), and National Institutes of Health Stroke Scale (NIHSS), alongside complementary measures including the Modified Barthel Index (MBI), Medical Research Council (MRC) scale, and neurophysiological or neuroimaging tools such as transcranial magnetic stimulation (TMS) and diffusion tensor imaging (DTI).

Across these studies, six reported statistically significant associations between the Met allele and poorer motor recovery [17,18,19,20,25,26]. These associations manifested as reduced improvement in FMA scores, higher mRS scores indicating greater disability, lower probability of achieving clinically meaningful motor gains, and diminished responsiveness to high-frequency rTMS protocols. In particular, individuals with the Val/Val genotype were more likely to demonstrate significant improvement following rTMS, whereas Met allele carriers exhibited comparatively attenuated functional gains. Two studies did not identify significant genotype-related differences in motor recovery [18,27], and one reported genotype-dependent differences in white matter integrity without corresponding differences in clinical motor scores [19].

Braun et al. [27] evaluated the influence of the BDNF Val66Met polymorphism on functional outcomes in young patients with first ischemic stroke, using the mRS. Carriers of the Met allele had a significantly higher risk of unfavorable outcomes (mRS ≥ 3, interpreted as loss of functional independence), suggesting that the polymorphism is associated with worse functional evolution even in younger populations. Mirowska-Guzel et al. [29], in a randomized, double-blind, placebo-controlled trial, found no significant differences in genotype distribution or rTMS efficacy, a result likely influenced by limited sample size. Similarly, Bembenek et al. [21] reported strong correlations between motor evoked potentials (MEPs) and upper-limb recovery but no association between BDNF polymorphisms and motor outcomes, again constrained by small cohort size.

Other studies highlighted genotype-modulated responsiveness to rTMS. Chang et al. [18,25] demonstrated that Val/Val homozygotes were more likely to achieve clinically significant improvement in upper-limb motor function, whereas Met carriers showed attenuated gains. Chang et al. [20] further identified the number of Met alleles, age, and initial FMA-UL score as independent predictors of motor outcome, with the polymorphism acting as a significant factor in patients with severe impairment. Kim et al. [17] suggested that Val66Met may be a prognostic factor in recovery, though their findings did not reach statistical significance. Neuroimaging studies by Kim et al. [19] and Park et al. [26] revealed genotype-dependent differences in corticospinal tract integrity and white matter microstructure, indicating distinct mechanisms of axonal degeneration, demyelination, and interhemispheric connectivity that may shape recovery trajectories.

Collectively, the majority of studies consistently reported less favorable motor outcomes among Met allele carriers during the acute and early subacute phases of stroke. Although effect measures and statistical models varied, the overall pattern supports a deleterious influence of the Val66Met polymorphism on motor recovery. These findings reinforce the biological plausibility that reduced activity-dependent BDNF secretion impairs corticospinal plasticity and limits responsiveness to rehabilitation interventions.

#### 4.1.2. Cortical Excitability and Neuroplasticity

Transcranial magnetic stimulation (TMS) is a non-invasive, painless, and effective method of modulating brain activity by inducing electrical currents through magnetic fields applied to the scalp. This technique can alter the excitability and plasticity of specific cortical neuronal populations, depolarizing neurons to produce action potentials and coordinating activity across cortical regions [30]. It has been widely studied in stroke research, with growing evidence of its effectiveness in promoting motor recovery [31].

Di Lazzaro et al. [24] conducted an observational study involving 20 patients in the acute phase of ischemic stroke. They found that Met allele carriers of the BDNF Val66Met polymorphism exhibited a significantly reduced interhemispheric imbalance in cortical excitability compared to patients with the Val/Val genotype. In contrast, Val/Val patients showed greater excitability in the unaffected hemisphere, suggesting that BDNF may influence interhemispheric reorganization following stroke. This investigation assessed resting motor threshold (RMT), active motor threshold (AMT), and interhemispheric imbalance measures. The attenuated imbalance observed among Met carriers suggests altered cortical reorganization dynamics. Although the clinical implications of reduced interhemispheric imbalance remain complex, these findings support the hypothesis that the Val66Met polymorphism modulates early cortical neuroplastic responses.

The biological rationale for this observation lies in BDNF’s role in synaptic potentiation and long-term plasticity. Reduced activity-dependent BDNF release in Met carriers may diminish the capacity for adaptive cortical reorganization following injury. Although limited to a single cohort, this mechanistic evidence strengthens the plausibility of genotype-dependent modulation of recovery observed in motor studies. Complementarily, studies in healthy individuals have shown that the Val66Met polymorphism is associated with diminished responses to TMS, including smaller increases in motor evoked potential amplitudes and less reorganization of motor maps after training, relative to non-carriers [32].

#### 4.1.3. Swallowing Function (Dysphagia)

Oropharyngeal dysphagia is a prevalent complication following a stroke, clinically manifesting as alterations in the transfer of food and/or liquids from the oral cavity to the stomach. Clinically, post-stroke dysphagia is associated with an increased risk of aspiration pneumonia, malnutrition, mortality, and other adverse functional outcomes [33]. In this context, recent studies have investigated the influence of genetic factors on swallowing recovery.

The prospective cohort study by Oh et al. [22], involving 206 patients, demonstrated that swallowing recovery after stroke may be modulated by the BDNF rs6265 polymorphism. Patients with the Val/Val genotype showed faster and more significant improvement on the Functional Oral Intake Scale (FOIS) and safer swallowing recovery than those with the Met allele. Notably, the proportion of patients transitioning from null oral intake to functional swallowing within the first month post-stroke was significantly higher among Val homozygotes. Improvements were documented across multiple swallowing scales, including FOIS and related assessments, reinforcing the potential role of BDNF in post-stroke bulbar recovery.

These findings suggest that the Val66Met polymorphism may influence neuroplastic reorganization within cortical and subcortical swallowing networks. Although evidence in this domain is currently limited to a single large cohort, the direction of effect aligns with the broader pattern observed in motor recovery, supporting the hypothesis of reduced plastic potential among Met carriers. Complementarily, Essa et al. [10] suggested that the Val66Met polymorphism may predict improvement in swallowing in response to pharyngeal electrical stimulation, indicating that genetic variability may influence both spontaneous recovery and responsiveness to therapeutic interventions.

#### 4.1.4. Language Function (Aphasia)

Aphasia is one of the main sequelae of stroke, characterized as an acquired language disorder that impairs the comprehension and/or production of oral and written language [34]. The BDNF gene polymorphism has been investigated as a potential factor influencing recovery from aphasia after stroke.

Mirowska-Guzel et al. [23] evaluated 20 patients with post-stroke aphasia and found that serum BDNF levels were significantly higher in non-rTMS control groups at both 6 h and 3 weeks after rehabilitation. Differences in BDNF concentrations were observed between men and women at different timepoints, suggesting sex-specific modulation of BDNF secretion. In men, a significant difference in BDNF concentrations was observed between rTMS-treated and placebo groups at the 6 h mark. In contrast, in women, this difference emerged only after 3 weeks, regardless of clinical improvement. Additionally, both age and rehabilitation duration significantly influenced BDNF levels in repeated-measures analyses. However, the study revealed no association between BDNF polymorphisms and serum protein levels, nor between genotype and clinical language outcomes. Similarly, De Boer et al. [12] reported no significant differences in language recovery between Met allele carriers and non-carriers. These findings are consistent with the broader observation that early-phase aphasia recovery did not show a statistically significant association with the Val66Met genotype, and that serum BDNF concentrations did not differ significantly by genotype status [23].

Interpretation of these findings should account for the relatively modest sample sizes and potential limitations in statistical power. Moreover, language recovery may engage compensatory bilateral network recruitment mechanisms distinct from corticospinal-dependent motor pathways, potentially attenuating detectable genotype effects. Therefore, although current evidence does not support a strong influence of Val66Met on early aphasia recovery, these conclusions remain provisional and warrant validation in larger, longitudinal cohorts with comprehensive neurophysiological and imaging-based assessments.

Language-related outcomes diverge from the other domains analyzed and are consistent with the literature, which indicates that, in the acute phase of aphasia, BDNF polymorphisms do not constitute a substantially determining factor for language recovery in recent stroke cases [35]. In contrast, evidence from patients in the chronic phase points to a more relevant role of this polymorphism, possibly due to the greater involvement of late neuroplasticity mechanisms. In this context, Dresang et al. [36] demonstrated that the BDNF polymorphism is a critical predictor of variability in aphasia severity in chronic individuals, with Val/Val genotype carriers presenting less severe impairments compared to Met allele carriers. These findings are consistent with those of Lee et al. [37], who also identified a significant association between the BDNF gene and language recovery, with the Met allele being associated with poorer functional outcomes.

Additionally, more recent evidence suggests a potential modulatory role of the BDNF Val66Met polymorphism in response to therapeutic interventions in post-stroke aphasia. The meta-analysis by Khan et al. [38] supports the effectiveness of transcranial electrical stimulation in improving aphasia and attentional deficits across different stages of stroke, raising the hypothesis that genetic factors, such as BDNF, may influence the magnitude of the therapeutic response. These findings highlight the importance of considering the interaction between time since lesion, neural plasticity mechanisms, and genetic profile when interpreting results and designing more individualized rehabilitation strategies.

### 4.2. Integrated Interpretation of Domain Findings

**Figure 2:**
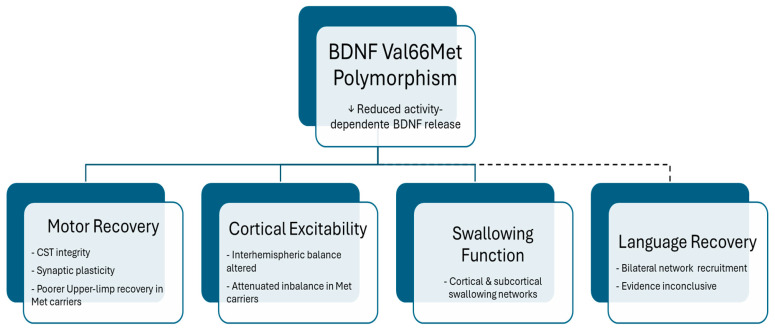
Mechanistic model of the BDNF Val66Met polymorphism in post-stroke recovery. The Val66Met polymorphism reduces activity-dependent BDNF release, thereby altering neuroplasticity. This genetic variation has been consistently associated with poorer outcomes in motor recovery, cortical excitability, and swallowing function, while evidence for language recovery remains inconclusive. Solid arrows indicate domains with unfavorable outcomes in Met carriers; dashed arrows indicate domains with uncertain associations. Solid arrows = consistent unfavorable outcomes in Met carriers (Motor, Excitability, Swallowing). Dashed arrow = inconclusive evidence (Language).

The most consistent associations were observed in motor recovery paradigms, particularly in studies assessing upper-limb function, global disability indices, and responsiveness to neuromodulatory interventions. Although swallowing and cortical excitability domains were each represented by a single cohort, their findings aligned directionally with the broader pattern of diminished recovery efficiency among Met allele carriers. In contrast, the language recovery domain remains insufficiently supported to establish a definitive genotype-dependent trend. Figure 2 summarizes these results.

### 4.3. Quality Assessment and Limitations of the Selected Articles

The Genetic Risk Prediction Studies (GRIPS) guideline was applied to assess the quality of the included studies using 20 of the 25 criteria. All eleven studies were classified as high quality, meeting at least 75% of the evaluated items, with five studies fulfilling all criteria [20,21,22,25,26].

Overall, the main methodological limitations were related to incomplete reporting of risk model validation, which was absent in more than half of the studies [17,18,19,23,24,27], and insufficient description of validation procedures [17,23,24,27]. Additional issues included lack of reporting on predicted risk distribution [17,18,24] and missing data handling [27].

Despite these limitations, most studies adequately reported key methodological components, supporting the overall robustness of the included evidence.

### 4.4. Limitations

Several limitations should be considered when interpreting these findings. Although all included studies reported allele and genotype frequencies, there was considerable variability in population characteristics and sample sizes, with some cohorts being notably small, which may limit the generalizability of the results. Additionally, there was a clear geographic imbalance, with a predominance of studies conducted in Eastern populations, particularly in Korea (7 out of 11 studies), which restricts the extrapolation of findings to other ethnic and regional groups.

Methodological differences, variation in outcome measures, and the limited number of available studies may also have influenced the conclusions. The search strategy was limited to English-language publications, potentially excluding relevant studies published in other languages.

Future studies should aim to further elucidate the role of BDNF polymorphisms in post-stroke recovery by adopting longitudinal designs that enable the investigation of their effects across different phases of stroke, including the acute, subacute, and chronic stages. Such approaches may help clarify the temporal dynamics of neuroplasticity and the extent to which genetic factors differentially influence recovery trajectories over time. In addition, studies should prioritize larger and more diverse populations, as well as the use of standardized outcome measures, to enhance comparability and generalizability of findings.

## 5. Conclusions

This systematic review suggests that the Val66Met polymorphism of the BDNF gene is associated with differences in post-stroke functional recovery, with the Met allele frequently linked to worse outcomes in motor recovery, brain excitability, swallowing function, and language. Despite this overall trend, some studies did not observe significant associations, underscoring the complexity of recovery mechanisms and the multifactorial nature of neuroplastic adaptation after stroke.

These findings suggest the potential of BDNF genotype as a prognostic biomarker, while also emphasizing the need for caution. Variability in study design, sample size, and population characteristics limits the ability to draw definitive conclusions. Future research should prioritize larger, multi-center cohorts across diverse ethnic groups, employ standardized methodologies, and incorporate longitudinal neuroimaging and neurophysiological assessments.

Such efforts are crucial for validating the role of the Val66Met polymorphism and advancing personalized rehabilitation strategies. Integrating genetic profiling into clinical practice may enable the tailoring of interventions, such as neuromodulatory therapies, to individual recovery trajectories, ultimately improving functional outcomes and reducing the burden of stroke-related disability.

## Data Availability

No new data were created or analyzed in this study. The data used in the review are those from the article’s bibliographic references. Articles that met the inclusion criteria were analyzed according to the Genetic Risk Prediction Studies (GRIPS) guideline to determine their quality. We used other bibliographic references only to discuss or clarify points from the selected articles presented in Table 1 and Table 2.

## Supplementary Materials

The following supporting information can be downloaded at: https://www.mdpi.com/article/10.3390/biomedicines14071637/s1. Table S1: PRISMA 2020 Checklist [39]; Table S2: Quality Evaluation of articles according to the adapted GRIPS guideline; Table S3. Studies excluded after full-text assessment and reasons for exclusion according to PRISMA criteria.
