# Supplementary material for: BDNF Val66met Gene Polymorphism in Primary Acute and Subacute Stroke Functional Recovery: A Systematic Review
Source: Biomedicines. 2026 Jul 20;14(7):1637. doi: 10.3390/biomedicines14071637 (PMC13407042; doi:10.3390/biomedicines14071637)
Supplement: Supplementary file 1 [file biomedicines-14-01637-s001.zip › Supplementary Table S2_GRIPS_BDNF and Stroke_MDPI Biomedicine.pdf]

biomedicines

MDPI

Systematic Review

BDNF Val66met Gene Polymorphism in Primary Acute and Subacute Stroke Functional Recovery: A Systematic Review

Juliana Moura Alves Seixas [1,2], Cristina Lemos Barbosa Furia [3], Matheus Gomes de Castro [1,2], Larissa Sousa Silva Bonasser [2,4], Ligia Canongia de Abreu Cardoso Duarte [1,2], Calliandra Maria de Souza Silva [1,3,5] and Izabel Cristina Rodrigues da Silva [1,2,3,4,*]

1 Postgraduate Program in Health Sciences and Technologies, Faculty of Health Sciences and Technologies, University of Brasília (UnB), Brasília 72220-900. Brazil; juliana.seixas@aluno.unb.br (J.M.A.S.); matheuscastrodf@gmail.com (M.G.d.C.); ligia.canongia@gmail.com (L.C.d.A.C.D.); cdssilva@gmail.com (C.M.d.S.S.)
2 Clinical Analysis Laboratory, Molecular Pathology Sector, Pharmacy Department, Faculty of Health Sciences and Technologies, University of Brasília (UnB), Brasília 72220-900, Brazil;
3 Faculty of Health Sciences and Technologies, University of Brasília (UnB), Brasília 72220-900, Brazil; furiacristina@gmail.com
4 Postgraduate Program in Health Sciences, University Campus Darcy Ribeiro, University of Brasília (UnB), Brasília 72220-900, Brazil
5 Academic Unit of Biotechnology Engineering (UAEB), Center for Sustainable Development of the Semi-Arid Region (CDSA), Sumé Campus, Federal University of Campina Grande (UFCG), Sumé-Paraiba 58540-000, PB, Brazil
* Correspondence: belbiomedica@gmail.com; Tel.: +55-(61)-3107-8400

Table S2: Quality Evaluation of articles according to the adapted GRIPS guideline.

Section/Topic		Mirowska-Guzel et al. 2013	Kim, et al 2013	Chang et al.; 2014	Di Lazzaro et al.; 2015	Kim, et al 2015	Chang et al.; 2016	Chang et al.; 2017	Park et al.; 2020	Bembenek et al.; 2020	Braun et al., (2020)	Oh et al.; 2021
**Methods**												
**Study design and setting**	4) Present key elements of study design early in the paper and	x	x	x	x	x	x	x	x	x	x	x

	describe the setting, locations, and relevant dates, including periods of recruitment, exposure, follow-up, and data collection.											
**Participants**	5) Describe eligibility criteria for participants, and sources and methods of selection of participants.	x	x	x	x	x	x	x	x	x	x	x
**Variables: Definition**	6) Clearly define all participant characteristics, risk factors and outcomes. Clearly define genetic variants using a widely-used nomenclature system.	x	x	x	x	x	x	x	x	x	x	x

	7) (a) Describe sources of data and details of methods of assessment (measurement) for each variable.	x	x	x	x	x	x	x	x	x	x	x
**Variables: Assessment**	(b) Give a detailed description of genotyping and other laboratory methods.	x	x	x	x	x	x	x	x	x	x	x
	8) (a) Describe how genetic variants were handled in the analyses	x	x	x	x	x	x	x	x	x	x	x
**Variables: Coding**	(b) Explain how other quantitative variables were handled in the analyses. If applicable, describe which groupings were	x	x	x	x	x	x	x	x	x	x	x

	chosen, and why.											
**Analysis: Risk model construction**	9) Specify the procedure and data used for the derivation of the risk model. Specify which candidate variables were initially examined or considered for inclusion in models. Include details of any variable selection procedures and other model building issues. Specify the horizon of risk prediction (e.g., 5-year risk).	x	x	x	x	x	x	x	x	x		x

**Analysis: Validation**	10) Specify the procedure and data used for the validation of the risk model.			x		x	x	x	x	x		x
**Analysis: Missing data**	11) Specify how missing data were handled.	x	x	x	x	x	x	x	x	x		x
**Analysis: Statistical methods**	12) Specify all measures used for the evaluation of the risk model including, but not limited to, measures of model fit and predictive ability.	x	X	x	x	x	x	x	x	x	x	x
**Analysis: Other**	13) Describe all subgroups, interactions, and exploratory analyses that were examined.	x	x	x	x	x	x	x	x	x	x	x
**Results**												

**Participants**	14) Report the numbers of individuals at each stage of the study. Give reasons for nonparticipation at each stage. Report the number of participants not genotyped, and reasons why they were not genotyped.	x	x	x	x	x	x	x	x	x	x	x
**Descriptives: Population**	15) Report demographic and clinical characteristics of the study population, including risk factors used in the risk modeling.	x	x	x	x	x	x	x	x	x	x	x
**Descriptives: Model estimates**	16) Report unadjusted associations between the variables in	x	X	x	x	x	x	x	x	x		x

	the risk model(s) and the outcome. Report adjusted estimates and their precision from the full risk model(s) for each variable.											
**Risk distributions**	17) Reports the predicted risk distribution and/or its scores.	x				x	x	x	x	x		x
**Assessment**	18) Report measures of model fit and predictive ability, and any other performance measures, if pertinent.	x	X	x	x	x	x	x	x	x		x
**Validation**	19) Report any validation of the risk model(s).						x	x	x	x		x

**Other analyses**	20) Present results of any subgroup, interaction, or exploratory analyses, whenever pertinent.	x	x	x	x	x	x	x	x	x	x	x
**Discussion**												
**Limitations**	21) Discuss limitations and assumptions of the study, particularly those concerning study design, selection of participants, and measurements and analyses, and discuss their impact on the results of the study.	x	x	x	x	x	x	x	x	x	x	x
**Interpretation**	22) Give an overall interpretation of results	x	x	x	x	x	x	x	x	x	x	x

	considering objectives, limitations, multiplicity of analyses, results from similar studies, and other relevant evidence.											
**Generalizability**	23) Discuss the generalizability and, if pertinent, the health care relevance of the study results.	x	x	x	x	x	x	x	x	x	x	x

Present: X.
