# Supplementary material for: BDNF Val66met Gene Polymorphism in Primary Acute and Subacute Stroke Functional Recovery: A Systematic Review
Source: Biomedicines. 2026 Jul 20;14(7):1637. doi: 10.3390/biomedicines14071637 (PMC13407042; doi:10.3390/biomedicines14071637)
Supplement: Supplementary file 1 [file biomedicines-14-01637-s001.zip › Supplementary Table S3_Studies excluded after full-text assessment and reasons for exclusion according to PRISMA criteria.pdf]

cells MDPI

**Table S3. Studies excluded after full-text assessment and reasons for exclusion according to PRISMA criteria**

| Authors | Year | Title | Reason for exclusion (full-text assessment) | PRISMA exclusion criterion |
|---|---|---|---|---|
| **Siironen et al.** | 2007 | The Met Allele of the BDNF Val66Met Polymorphism Predicts Poor Outcome Among Survivors of Aneurysmal Subarachnoid Hemorrhage | Not a stroke population (aneurysmal subarachnoid hemorrhage) | Wrong Participants |
| **Kim et al.** | 2008 | BDNF genotype potentially modifying the association between incident stroke and depression | Community-based longitudinal study, not focused on primary acute stroke sample | Wrong Participants |
| **Vilkki et al.** | 2008 | Relationship of the Met allele of the BDNF Val66Met polymorphism to memory after aneurysmal subarachnoid hemorrhage | Not a stroke population (subarachnoid hemorrhage) | Wrong Participants |
| **Zhou et al.** | 2011 | Decreased serum brain-derived neurotrophic factor (BDNF) is associated with post-stroke depression | Included post-stroke depression outcomes up to 6 months. | Wrong Participants |
| **Kim et al.** | 2011 | Serotonergic and BDNF genes and risk of depression after stroke | Study focused on post-stroke depression; population not restricted to primary stroke | Wrong Participants |
| **Manso et al.** | 2011 | Evidence for epistatic gene interactions between growth factor genes in stroke outcome | Did not analyze BDNF Val66Met polymorphism; epistatic analysis | Wrong Outcomes |

| | | | | |
|---|---|---|---|---|
| **Kim et al.** | 2012 | Associations of BDNF Genotype and Promoter Methylation with Acute and Long-Term Stroke Outcomes in an East Asian Cohort | Included long-term outcomes (1 year post-stroke), not restricted to primary acute stroke | Wrong Participants |
| **Mirowska-Guzel et al.** | 2012 | BDNF −270 C>T polymorphisms might be associated with stroke type and early neurological deficit | Genetic association study including hemorrhagic and ischemic stroke, not limited to primary acute stroke population. | Wrong Participants |
| **Cramer et al.** | 2012 | Correlation between genetic polymorphisms and stroke recovery | Study population not clearly restricted to first-ever (primary) stroke patients. | Wrong Participants |
| **Kim et al.** | 2013 | A longitudinal study of BDNF promoter methylation and genotype with poststroke depression | Outcomes assessed at two weeks and one year after stroke, not restricted to primary acute stroke phase | Wrong Participants |
| **Zhao et al.** | 2013 | Brain-derived neurotrophic factor G196A polymorphism predicts 90-day outcome of ischemic stroke in Chinese | Outcome assessed at 90 days post-stroke | Wrong Participants |
| **Kim et al.** | 2013 | A longitudinal study of BDNF promoter methylation and genotype with poststroke depression | Study focused on post-stroke depression; not restricted to primary stroke and not focused on Val66Met only | Wrong Participants / Incomplete Data |
| **Stanne et al.** | 2014 | Genetic Variation at the BDNF Locus: Evidence for Association with Long-Term Outcome after Ischemic Stroke | Long-term post-stroke outcomes; population not restricted to first-ever stroke | Wrong Participants |
| **Mirowska-Guzel et al.** | 2014 | Impact of BDNF −196 G>A and −270 C>T polymorphisms on stroke rehabilitation outcome | Rehabilitation-phase stroke population; primary stroke not specified | Wrong Participants |

| | | | | |
|---|---|---|---|---|
| **Uhm et al.** | 2015 | BDNF genotype influence the efficacy of rtms in stroke patients | Included only chronic stroke patients undergoing neuromodulation | Wrong Participants |
| **Lu et al.** | 2015 | Impact of repetitive transcranial magnetic stimulation on post-stroke dysmnesia | Post-stroke cognitive intervention; primary stroke not specified | Wrong Participants |
| **Liepert et al.** | 2015 | Polymorphismus des brain derived neurotrophic factor und Erholung nach Schlaganfall | Foreign language (non-English publication) | Foreign language |
| **Keshavarz et al.** | 2016 | Association of BDNF G196A Gene Polymorphism with Ischemic Stroke Occurrence and its 6-Month Outcome in an Iranian Population | Follow-up outcomes assessed months after stroke event (chronic phase) | Wrong Participants |
| **Rezaei et al.** | 2016 | Brain-derived neurotrophic factor (BDNF) Val66Met polymorphism and post-stroke dementia | Outcomes assessed months after stroke (post-stroke dementia) | Wrong Participants |
| **Helm et al.** | 2016 | The presence of a single nucleotide polymorphism in the BDNF gene affects the rate of locomotor adaptation after stroke | Study conducted exclusively in chronic stroke survivors (>6 months) | Wrong Participants |
| **Niimi et al.** | 2016 | Role of Brain-Derived Neurotrophic Factor in Beneficial Effects of Repetitive Transcranial Magnetic Stimulation for Upper Limb Hemiparesis after Stroke | Incomplete Val/Met genotype data | Incomplete Data |
| **Van der Vliet et al.** | 2017 | BDNF Val66Met but not transcranial direct current stimulation affects motor learning after stroke | Included only chronic stroke patients | Wrong Participants |

| | | | | |
|---|---|---|---|---|
| **Essa et al.** | 2017 | The BDNF polymorphism Val66Met may be predictive of swallowing improvement post pharyngeal electrical stimulation in dysphagic stroke patients | Intervention study including patients up to 6 weeks post-stroke, not restricted to primary stroke | Wrong Participants |
| **De Boer et al.** | 2017 | The Role of the BDNF Val66Met Polymorphism in Recovery of Aphasia After Stroke | Inpatient rehabilitation population with post-stroke aphasia. Study population not clearly restricted to first-ever (primary) stroke patients. | Wrong Participants |
| **Charalambous et al.** | 2018 | A single exercise bout and locomotor learning after stroke: physiological, behavioural, and computational outcomes | Study conducted exclusively in individuals with chronic stroke (>3 months post-stroke) | Wrong Participants |
| **Fridriksson et al.** | 2018 | BDNF genotype and tdcs interaction in aphasia treatment | Study conducted in individuals with chronic post-stroke aphasia. | Wrong Participants |
| **French et al.** | 2018 | The relationship between BDNF Val66Met polymorphism and functional mobility in chronic stroke survivors | Chronic stroke population (>6 months post-stroke) | Wrong Participants |
| **Liang et al.** | 2018 | Genetic variations in the p11/tPA/BDNF pathway are associated with post-stroke depression | Did not focus exclusively on BDNF Val66Met polymorphism | Wrong Outcomes |
| **Kovaleva et al.** | 2018 | Correlation of the genetic profile and features of the rehabilitation after ischemic stroke | Foreign language and did not evaluate BDNF Val66Met polymorphism | Foreign language |

| | | | | |
|---|---|---|---|---|
| **Cattagni et al.** | 2019 | A single session of anodal transcranial direct current stimulation applied over the affected primary motor cortex does not alter gait parameters in chronic stroke survivors | Included only chronic stroke survivors | Wrong Participants |
| **Zhou et al.** | 2019 | Differences in brain-derived neurotrophic factor gene polymorphisms between acute ischemic stroke patients and healthy controls | Case-control genetic association study; stroke population not clearly restricted to first-ever (primary) stroke patients. | Wrong Participants |
| **Rezaei et al.** | 2020 | BDNF (rs6265) Val<Met polymorphism can buffer cognitive functions against post stroke CT/MRI pathological findings | Post-stroke cognitive outcomes assessed after the acute phase | Wrong Participants |
| **Santoro et al.** | 2020 | BDNF rs6265 Polymorphism and Its Methylation in Patients with Stroke Undergoing Rehabilitation | Study conducted during stroke rehabilitation phase, including subacute/chronic patients | Wrong Participants |
| **Park et al.** | 2020 | Differential Relationship between Microstructural Integrity in White Matter Tracts and Motor Recovery following Stroke Based on BDNF Genotype | Motor recovery assessed up to 3 months post-stroke | Wrong Participants |
| **Han et al.** | 2020 | BDNF Met allele is associated with lower cognitive function in poststroke rehabilitation | Included first or recurrent stroke patients during rehabilitation | Wrong Participants |
| **Sang et al.** | 2020 | Influence of acupuncture on patients with post-stroke depression | Post-stroke depression population up to 1 year after stroke | Wrong Participants |
| **Rezaei et al.** | 2020 | Significant Destructive Interaction of BDNF Val66Met Polymorphism with Stroke Severity and Family History of Dementia for Cognitive Impairments | Case-control study with chronic post-stroke population (~200 days post-stroke) | Wrong Participants |

| | | | | |
|---|---|---|---|---|
| **Parchure et al.** | 2022 | BDNF Gene Polymorphism Predicts Response to Continuous Theta Burst Stimulation in Chronic Stroke Patients | Included only chronic stroke patients | Wrong Participants |
| **Dresang et al.** | 2022 | Genetic and neurophysiological biomarkers of neuroplasticity inform post-stroke language recovery | Study conducted in chronic post-stroke aphasia population (>3 months) | Wrong Participants |
| **Park et al.** | 2022 | Single Nucleotide Polymorphisms May Increase the Risk of Aspiration Pneumonia in Post-Stroke Patients with Dysphagia | Did not focus on BDNF Val66Met polymorphism | Wrong Outcomes / Incomplete Data |
| **Cramer et al.** | 2022 | Genetic Factors, Brain Atrophy, and Response to Rehabilitation Therapy after Stroke | Rehabilitation-phase population; mixed stroke history. Incomplete genetic data; BDNF Val66Met genotype frequencies not reported. | Incomplete Data |
| **Sadhukhan et al.** | 2023 | Genetic Variations and Altered Blood mrna Level of Circadian Genes and BDNF as Risk Factors of Post-Stroke Cognitive Impairment | Post-stroke cognitive impairment population beyond acute phase | Wrong Participants |
| **Aldridge et al.** | 2023 | Single Nucleotide Polymorphisms Associated With Motor Recovery in Patients With Nondisabling Stroke | Genome-wide association study not focused on the BDNF Val66Met polymorphism | Wrong Outcomes |
| **Nair et al.** | 2024 | Genetic variants in BDNF (rs6265 and rs11030119) and stroke susceptibility | Case-control genetic susceptibility study, not focused on acute stroke patients | Wrong Participants |
| **Ai et al.** | 2024 | Interactions between tdcs treatment and COMT Val158Met in poststroke cognitive impairment | Poststroke cognitive impairment population; primary stroke not specified | Wrong Participants |

| | | | | |
|---|---|---|---|---|
| **Cramer et al.** | 2024 | Genetic Variation and Stroke Recovery: The STRONG Study | Did not provide complete Val/Met genotype distribution for BDNF Val66Met | Incomplete Data |
